# GNPAT-driven plasmalogen biosynthesis regulates macrophage polarization and protects against obesity-induced inflammation by suppressing PKCδ–STAT1 signaling

**DOI:** 10.1016/j.molmet.2026.102440

**Published:** 2026-09-18

**Authors:** Ji-Young Yun, Myoung Seok Ko, Chung Hwan Hong, Ji Eun Yoon, Jaechan Leem, Mi-Ok Kim, Su Jung Kim, Hyun Ju Yoo, Yun-kyung Cho, In-Jeoung Baek, Young-Bum Kim, Ki-Up Lee, Eun Hee Koh

**Affiliations:** 1 Biomedical Research Center, Asan Institute for Life Sciences, Asan Medical Center, University of Ulsan College of Medicine, Seoul, South Korea; 2 Department of Medical Science, Asan Medical Center, University of Ulsan College of Medicine, Seoul, South Korea; 3 Department of Internal Medicine, Asan Medical Center, University of Ulsan College of Medicine, Seoul, South Korea; 4 ConveRgence mEDIcine research cenTer (CREDIT), Asan Institute for Life Sciences, Asan Medical Center, University of Ulsan College of Medicine, Seoul, South Korea; 5 Department of Convergence Medicine and Asan Institute for Life Sciences, Asan Medical Center, University of Ulsan College of Medicine, Seoul, South Korea; 6 Department of Medicine, Division of Endocrinology, Diabetes, and Metabolism, Beth Israel Deaconess Medical Center, Harvard Medical School, Boston, MA, USA

**Keywords:** Adipose tissue, Glyceronephosphate O-acyltransferase (GNPAT), Insulin resistance, Macrophage, Plasmalogens, Protein kinase Cδ (PKCδ), Signal transducer and activator of transcription 1 (STAT1)

## Abstract

Chronic inflammation in white adipose tissue (WAT) contributes to obesity-associated insulin resistance. Plasmalogens, ether phospholipids synthesized in peroxisomes, have been implicated in immune regulation. GNPAT (glyceronephosphate O-acyltransferase) is a peroxisomal enzyme that catalyzes the first and rate-limiting step of plasmalogen biosynthesis. Here, we show that dietary supplementation with alkyl glycerol (AG), a plasmalogen precursor, improves insulin sensitivity and reduces adipose inflammation in high-fat diet (HFD)-fed mice, independent of changes in body weight or invariant natural killer T cell activation. AG treatment reduced M1-like macrophage infiltration and proinflammatory gene expression in WAT without altering M2 macrophage polarization. Furthermore, AG supplementation restored levels of docosahexaenoic acid-enriched plasmalogen species that were reduced by HFD. In contrast, *Gnpat*^+^/^−^ mice displayed impaired plasmalogen biosynthesis, elevated M1 polarization, and exacerbated insulin resistance upon HFD feeding. Mechanistically, GNPAT overexpression in macrophages inhibited protein kinase C delta (PKCδ) activation and downstream signal transducer and activator of transcription 1 (STAT1) signaling, attenuating lipopolysaccharide-induced inflammation. *In vivo* studies showed that AG supplementation reduced diacylglycerol accumulation, suggesting that altered lipid flux contributes to the modulation of PKCδ signaling observed in macrophages. Of translational relevance, GNPAT expression was decreased in the visceral adipose tissue of humans with type 2 diabetes. These findings establish GNPAT-driven plasmalogen biosynthesis as a key modulator of adipose immune homeostasis via suppression of PKCδ–STAT1 signaling. Thus, enhancing plasmalogen levels may have therapeutic potential for obesity-related metabolic disorders.

## Highlights

• Alkyl glycerol (AG) supplementation improved insulin sensitivity in mice fed a high-fat diet.
• AG treatment reduced M1 macrophage infiltration and the expression of proinflammatory genes in adipose tissue.
• AG restored DHA-enriched plasmalogen species reduced by high-fat diet in white adipose tissue.
• GNPAT overexpression in macrophages suppressed PKCδ–STAT1 signaling and LPS-induced inflammation.
• GNPAT expression was decreased in the visceral adipose tissue of individuals with type 2 diabetes.

## Graphical abstract

**Figure:**
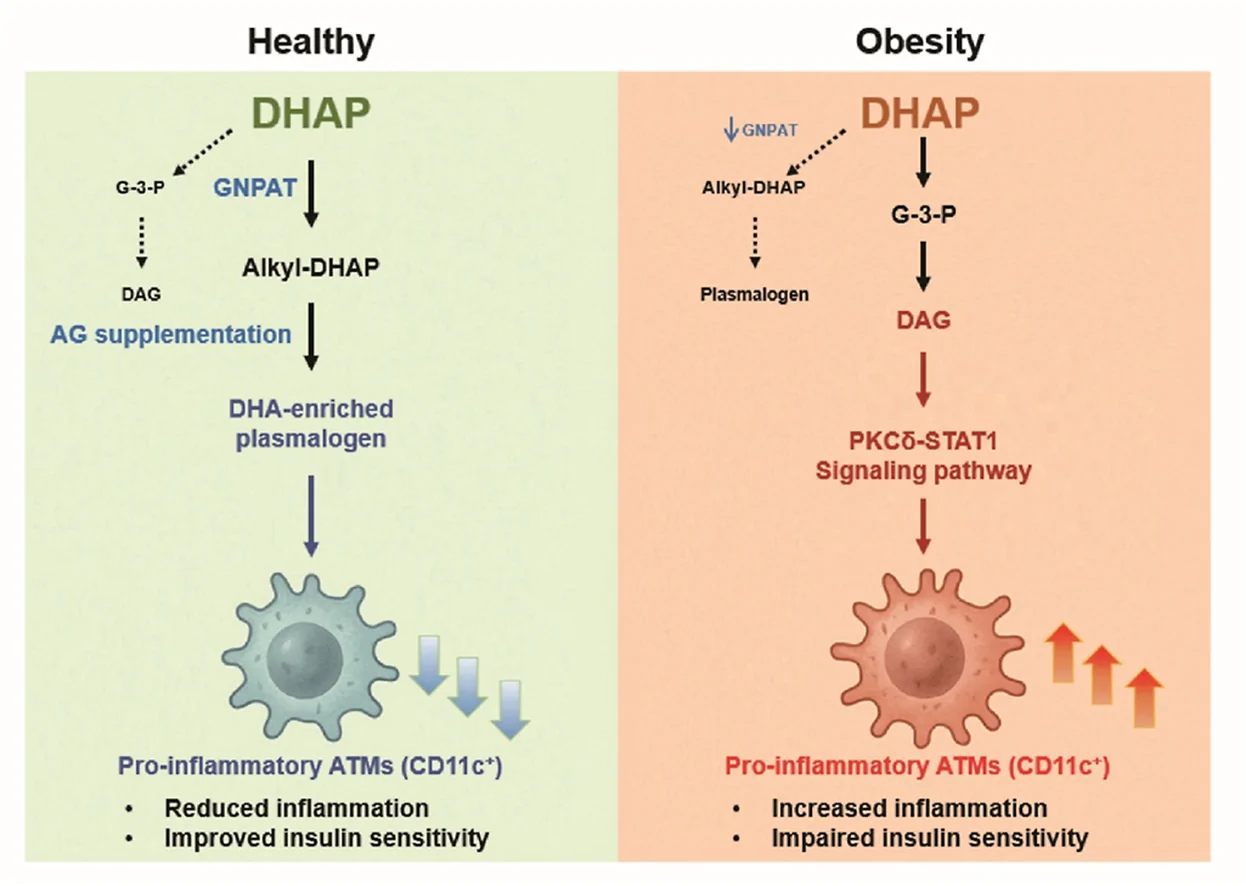

Dihydroxyacetone phosphate (DHAP) is converted to alkyl-DHAP by the enzyme glyceronephosphate O-acyltransferase (GNPAT). When alkyl glycerol (AG) is supplied, the production of plasmalogen is further enhanced. Moreover, AG treatment leads to the generation of docosahexaenoic acid (DHA)-enriched plasmalogen, which suppresses pro-inflammatory adipose tissue macrophage (ATM) polarization, thereby improving insulin sensitivity. However, in obesity, due to the downregulation of GNPAT, DHAP is instead converted to glycerol-3-phosphate (G-3-P), which is then metabolized to diacylglycerol (DAG). The accumulation of DAG activates the PKCδ-STAT1 signaling pathway, promoting the polarization of macrophages toward the pro-inflammatory M1 phenotype (CD11c+). This inflammatory response ultimately induces insulin resistance.

## 1 Introduction

Growing evidence indicates that low-grade inflammation in adipose tissue is a major contributor to the development of insulin resistance associated with obesity [[1], [2], [3]]. This condition predisposes individuals to various metabolic diseases, including atherosclerosis [4] and metabolic dysfunction-associated steatohepatitis (MASH) [1]. Among the diverse types of immune cells, adipose tissue macrophages (ATMs) play a central role in initiating adipose tissue inflammation and obesity-related insulin resistance [5,6]. In particular, high-fat diet (HFD) feeding promotes a phenotypic shift in ATMs from an anti-inflammatory (M2-like) to a proinflammatory (M1-like) state, thereby contributing to systemic insulin resistance [7,8]. Numerous studies have focused on elucidating the mechanisms by which macrophage activation drives adipose tissue inflammation and metabolic dysfunction in obesity [5,[9], [10], [11]].

Plasmalogens are a unique class of peroxisome-derived phospholipids characterized by an ether bond at the *sn*-1 position of the glycerol moiety [12], and serve as structural components of membranes, reservoirs for second messengers, and antioxidants [12,13]. Plasmalogens are primarily synthesized in the liver and transported to other tissues via lipoproteins [14]. They are highly abundant in nervous tissues, and defects in plasmalogen biosynthesis are associated with several inherited neurological diseases in humans [13,15,16]. In addition, recent studies have suggested that alterations in plasmalogen levels may be associated with metabolic and inflammatory diseases [13,[16], [17], [18]]. We previously demonstrated that hepatic plasmalogens, particularly those enriched with docosahexaenoic acid (DHA), protect against MASH by maintaining peroxisome proliferator-activated receptor α activity and enhancing fatty acid oxidation [18], highlighting their therapeutic potential in metabolic disorders.

Clinical and lipidomic studies have suggested a potential link between reduced plasmalogen levels and metabolic dysfunction. Low-density lipoprotein from obese patients with metabolic syndrome and type 2 diabetes mellitus showed decreased plasmalogen levels and increased lipid peroxidation compared to control volunteers [19]. Small high-density lipoprotein (HDL) particles predominated in dyslipidemic diabetic subjects and were associated with a low plasmalogen/Apolipoprotein A-I ratio [20]. Similarly, in a lipidomic study of twin pairs discordant for obesity, morbidly obese subjects showed markedly decreased plasmalogen levels in adipose tissue [21]. While these findings suggest that low plasmalogen levels may contribute to the pathogenesis of metabolic diseases and oxidative stress, HDL-associated plasmalogens increased in short-term overfed healthy humans [22], raising the possibility that changes in plasmalogen levels may be either causal or compensatory in obesity-related metabolic diseases.

In this study, we identified glyceronephosphate O-acyltransferase (GNPAT)-dependent plasmalogen biosynthesis as a key regulator of adipose tissue inflammation and systemic insulin sensitivity under dietary stress. Alkyl glycerol (AG) supplementation alleviated inflammation and improved metabolic outcomes, whereas *Gnpat* haploinsufficiency exacerbated immune and metabolic dysfunction. At the molecular level, GNPAT exerted its protective effects by suppressing protein kinase C delta (PKCδ)-mediated activation of signal transducer and activator of transcription 1 (STAT1) in macrophages. Together, these findings highlight the critical role of plasmalogen homeostasis in regulating metabolic and immune responses in obesity.

## 2 Results

### 2.1 AG improves systemic insulin sensitivity and glucose homeostasis in HFD-fed mice independent of adiposity

AG, a plasmalogen precursor independent of peroxisomal function (Figure 1A) [12], has been shown to restore tissue plasmalogen levels and prevent the progression of target-organ pathology in a plasmalogen-deficient mouse model [23]. To investigate the metabolic effects of plasmalogen, we administered AG to mice fed HFD. Body weight gain and the weight of epididymal WAT (eWAT) were not affected by AG in HFD-fed mice (Figure 1B,C). However, AG reduced plasma insulin levels without altering fasting glucose, indicating improved insulin sensitivity (Figure 1D,E). Consistent with this, AG improved glucose intolerance and insulin resistance in HFD-fed mice (Figure 1F,G), as evidenced by decreased AUC (area under the curve) during the glucose tolerance test (GTT) and insulin tolerance test (ITT), indicating a protective effect of AG on diet-induced metabolic dysfunction. To determine whether the reduction in insulin levels was due to impaired insulin secretion in response to AG, we performed an in vivo glucose-stimulated insulin secretion (GSIS) assay. AG-treated HFD-fed mice exhibited glucose-stimulated insulin responses indistinguishable from those of HFD controls (Figure 1H), indicating preserved β-cell secretory function. Thus, the reduction in insulin levels is unlikely to result from defective insulin secretion but instead reflects improved systemic insulin sensitivity. AG supplementation did not affect glucose tolerance or insulin sensitivity in chow-fed mice (Supplementary Fig. 1). To further evaluate its therapeutic potential, AG was administered after 4 weeks of HFD feeding, when insulin resistance was already established. Under these conditions, AG remained effective in improving glucose homeostasis (Supplementary Fig. 2A), indicating that its metabolic benefits extend beyond prevention to the treatment of established insulin resistance.

**Figure 1:**
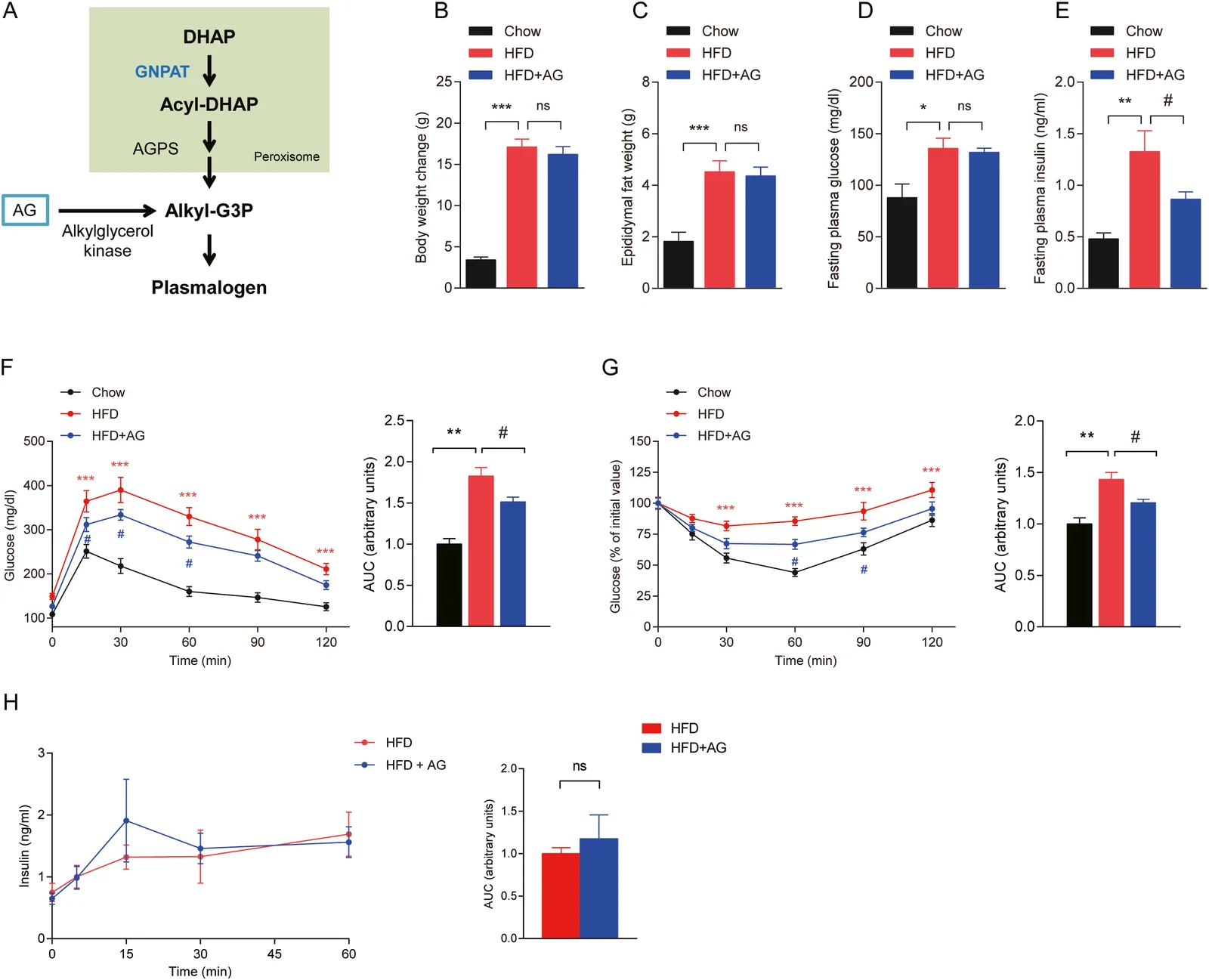
**Plasmalogen precursor AG alleviates diet-induced insulin resistance**. (A) Schematic diagram of plasmalogen biosynthesis. AGPS: Alkylglyceronephosphate synthase; Alkyl-G3P: Alkyl-glycerol-3-phosphate; DHAP: Dihydroxyacetone phosphate. (B–H) Mice were fed chow, HFD, or HFD + AG for 8 weeks. (B) Body weight. (C) Epididymal fat weight. (D) Fasting plasma glucose. (E) Fasting plasma insulin. (F) GTT and area under the curve (AUC) analysis. (G) ITT and AUC analysis. (H) GSIS and AUC analysis. Data are presented as mean ± SEM (n = 5). ∗*P* < 0.05, ∗∗*P* < 0.01, and ∗∗∗*P* < 0.005 versus chow group; ^#^*P* < 0.05 versus HFD group; ns, not significant.

To investigate the mechanisms underlying the AG-mediated insulin-sensitizing effect, we examined insulin signaling in skeletal muscle, a primary site of insulin-stimulated glucose disposal. HFD feeding impaired insulin-stimulated phosphorylation of Akt, whereas AG treatment restored this activation (Supplementary Fig. 3A). To determine whether AG directly improves insulin responsiveness in skeletal muscle cells, we examined insulin signaling and GLUT4 translocation in differentiated C2C12 myotubes following palmitic acid (PA)-induced insulin resistance. PA treatment markedly attenuated insulin-stimulated Akt phosphorylation, whereas AG treatment significantly restored Akt activation (Supplementary Fig. 3B). We next examined GLUT4 translocation, a key downstream event linking insulin signaling to glucose transport in skeletal muscle [24]. Insulin stimulation increased GLUT4 abundance in the plasma membrane fraction of control myotubes, whereas this response was markedly impaired by PA treatment. AG treatment restored insulin-stimulated GLUT4 accumulation in the plasma membrane fraction (Supplementary Fig. 3C). Together, these findings demonstrate that AG restores insulin signaling and downstream GLUT4 translocation in insulin-resistant skeletal muscle cells, supporting improved insulin-responsive glucose transport.

We next assessed whether AG directly modulates insulin signaling in adipocytes. In differentiated 3T3-L1 adipocytes, PA suppressed insulin-stimulated phosphorylation of Akt, consistent with an insulin-resistant state, whereas co-treatment with AG restored insulin signaling (Supplementary Fig. 4A). These effects occurred without significant changes in the expression of genes involved in de novo lipogenesis (*Fasn, Acly, Srebp1c*), fatty acid oxidation (*Cpt1a*), or lipolysis (*Atgl, Hsl*) in adipocytes (Supplementary Fig. 4B). Collectively, these findings demonstrate that AG improves systemic insulin sensitivity by enhancing insulin signaling in peripheral metabolic tissues, independent of changes in adiposity, and exerts both preventive and therapeutic effects in HFD-induced insulin resistance.

### 2.2 Improvement of insulin resistance by AG is independent of iNKT cell activation

Invariant natural killer T (iNKT) cells are recognized as a subset of type 2 inflammatory cells [25] and serve as key players in inflammation and dynamic changes in WAT [26,27]. Plasmalogen is essential for the development of iNKT cells, which play a key role in regulating inflammatory responses and are critical for mediating anti-inflammatory effects in obesity [28,29]. To investigate whether the metabolic effects of AG involve iNKT cell activation, we studied *Cd1d*^*−/−*^ mice, which lack iNKT cells due to the essential role of CD1d in the development and function of iNKT cells [28].

Consistent with previous reports [26], HFD reduced the number of iNKT cells, while AG treatment restored their levels (Supplementary Fig. 5A and B). However, *Cd1d*^*−/−*^ mice retained the effect of AG on HFD-induced insulin resistance (Supplementary Fig. 5C and D). These results suggest that the effect of AG on adipose tissue insulin resistance is independent of iNKT cell activation.

### 2.3 AG supplementation ameliorates adipose tissue inflammation and restores plasmalogen homeostasis in obesity

Adipose tissue inflammation is a primary causal factor in obesity-induced insulin resistance [30,31]. To evaluate the anti-inflammatory effects of AG, we examined adipose tissue histology and inflammatory gene expression. H&E staining demonstrated that AG treatment significantly reduced immune cell infiltration in adipose tissue (Figure 2A). Furthermore, immunohistochemical staining for F4/80, a marker specific to mature macrophages, showed increased macrophage accumulation in HFD-fed mice, which was markedly reduced by AG treatment (Figure 2A). Consistently, mRNA levels of proinflammatory markers, including F4/80, CD11c, *Tnf*, *Il-6*, and *Ccl2*, were significantly decreased by AG treatment in HFD-fed mice (Figure 2B), indicating that AG suppresses macrophage-driven adipose tissue inflammation. Importantly, this anti-inflammatory effect was also observed when AG was administered after insulin resistance had developed (Supplementary Fig. 2B).

**Figure 2:**
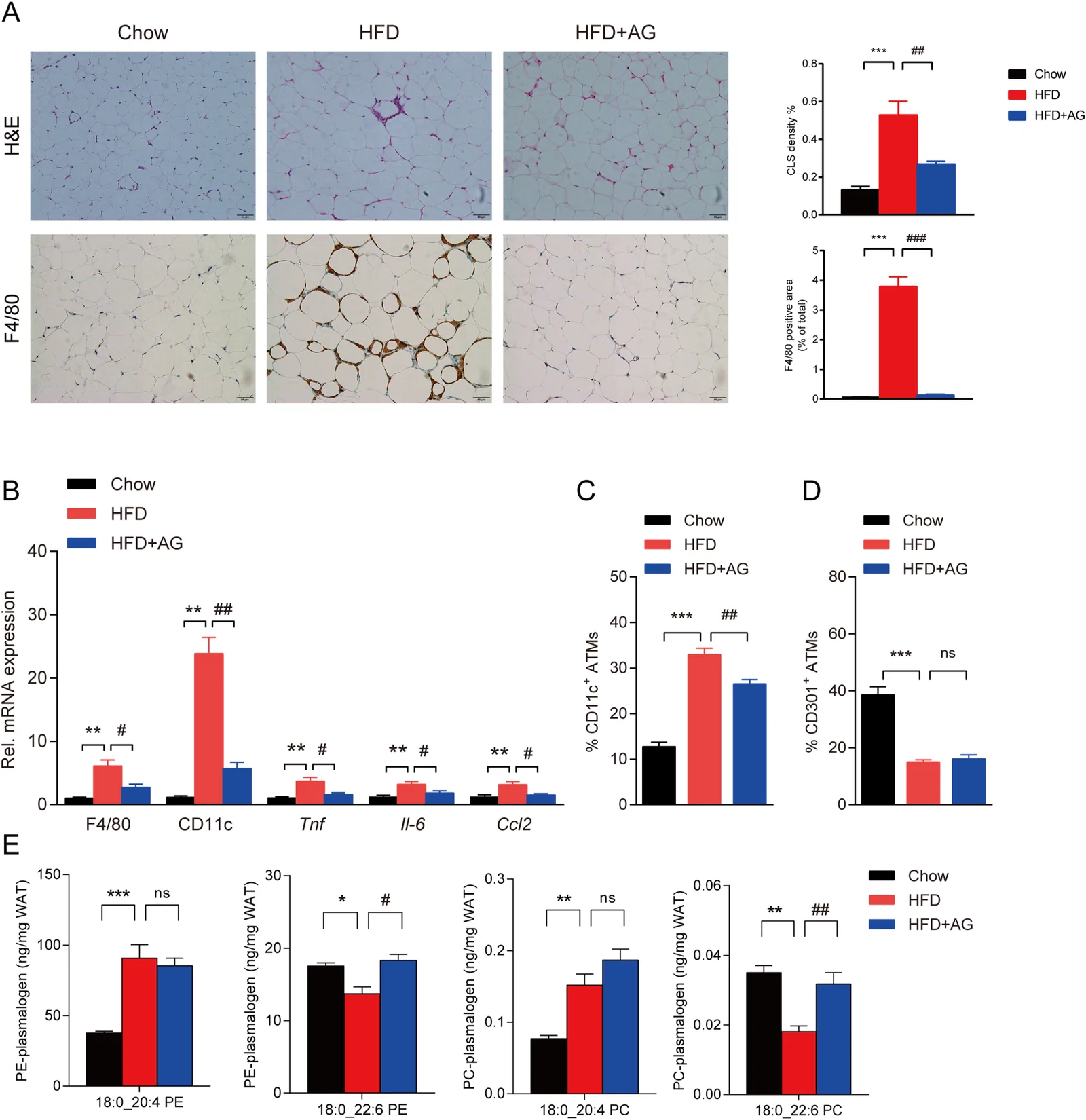
**AG selectively suppresses M1 macrophage accumulation without promoting the M2 phenotype**. (A) Representative H&E staining and F4/80 immunohistochemical staining of eWAT, and quantification of CLS density and F4/80-positive area. Scale bars, 50 μm. (B) Relative mRNA expression of *F4/80,* CD11c*, Tnf, Il-6*, and *Ccl2* in mice fed chow, HFD, or HFD + AG. (C, D) Percentage of ATMs stained as CD11b^+^F4/80^+^CD11c^+^ SVCs (C) and CD11b^+^F4/80^+^CD301^+^ (D) by FACS analysis. (E) Lipidomic analysis of PE- and PC-plasmalogen species in eWAT from mice fed chow or HFD, with or without AG supplementation (HFD + AG). Data are presented as mean ± SEM (n = 5–10). ∗*P* < 0.05, ∗∗*P* < 0.01, and ∗∗∗*P* < 0.005 versus chow group; ^#^*P* < 0.05, ^##^*P* < 0.01, and ^###^*P* < 0.005 versus HFD group; ns, not significant.

Flow cytometry analysis further confirmed the anti-inflammatory effects of AG on ATMs in eWAT (Supplementary Fig. 6A). Consistent with previous reports, HFD feeding increased the proportion of proinflammatory CD11c^+^ (M1-like) macrophages, accompanied by elevated CD11c mRNA expression [7]. AG treatment significantly reduced this M1-like macrophage population (Figure 2C). However, the proportion of anti-inflammatory, alternatively activated macrophages (M2) [32] was not significantly altered following AG treatment (Figure 2D). Interestingly, while AG effectively suppressed inflammation in eWAT, these anti-inflammatory effects were not observed in inguinal WAT (iWAT) (Supplementary Fig. 6B).

Taken together, these results suggest that AG supplementation ameliorates obesity-associated adipose tissue inflammation by reducing M1-like macrophage polarization rather than promoting M2-like phenotypes in eWAT.

To investigate how dietary conditions influence plasmalogen levels in adipose tissue, we performed targeted lipidomic profiling of eWAT from mice fed chow, HFD, and HFD supplemented with AG. Plasmalogens, which contain either ethanolamine (phosphatidylethanolamine (PE)-plasmalogen) or choline (phosphatidylcholine (PC)-plasmalogen) in their polar head groups, are enriched with polyunsaturated fatty acids (PUFAs), specifically arachidonic acid (AA, 20:4n-6) or DHA (22:6n-3) at the *sn*-2 position [18,33]. In the eWAT of HFD-fed mice, AA content within PE and PC plasmalogens was significantly increased, while DHA levels were markedly decreased (Figure 2E). Notably, AG treatment restored the DHA-enriched plasmalogen species that were diminished by HFD. In contrast, the elevated AA content remained unchanged following AG supplementation (Figure 2E). These findings suggest that AG restores DHA incorporation into adipose plasmalogens without affecting AA enrichment, thereby potentially supporting adipose immune–metabolic homeostasis under dietary stress and, at least in part, highlighting the beneficial effects of DHA on metabolic disturbances.

### 2.4 Downregulation of GNPAT in mouse and human adipose tissue is associated with HFD-induced inflammation and M1 macrophage polarization

GNPAT is the first and rate-limiting enzyme in the plasmalogen biosynthesis pathway, localized in peroxisomes [13,33]. To determine whether GNPAT plays a critical role in adipose tissue under metabolic stress, we first examined its expression in response to HFD feeding. As expected, *Gnpat* mRNA expression was significantly reduced in the eWAT of HFD-fed mice, including both adipocyte and stromal vascular fractions (SVF) (Figure 3A,B). To assess the clinical relevance of this finding, we further analyzed publicly available transcriptomic data from human omental adipose tissue (Gene Expression Omnibus (GEO): GSE16415). GNPAT expression was significantly lower in women with both obesity and type 2 diabetes (BMI >30 kg/m^2^, age >55 years) compared to age- and BMI-matched obese women with normal glucose tolerance (Figure 3C). To further strengthen the translational relevance of these findings, we analyzed an additional publicly available human omental adipose tissue transcriptomic dataset (GSE20950). *GNPAT* expression was significantly lower in insulin-resistant individuals than in insulin-sensitive individuals (Supplementary Fig. 7A), whereas *CCL2* expression was significantly increased in the insulin-resistant group (Supplementary Fig. 7B). These findings further demonstrate that reduced adipose GNPAT expression occurs in the setting of insulin resistance and increased adipose inflammatory gene expression in humans.

**Figure 3:**
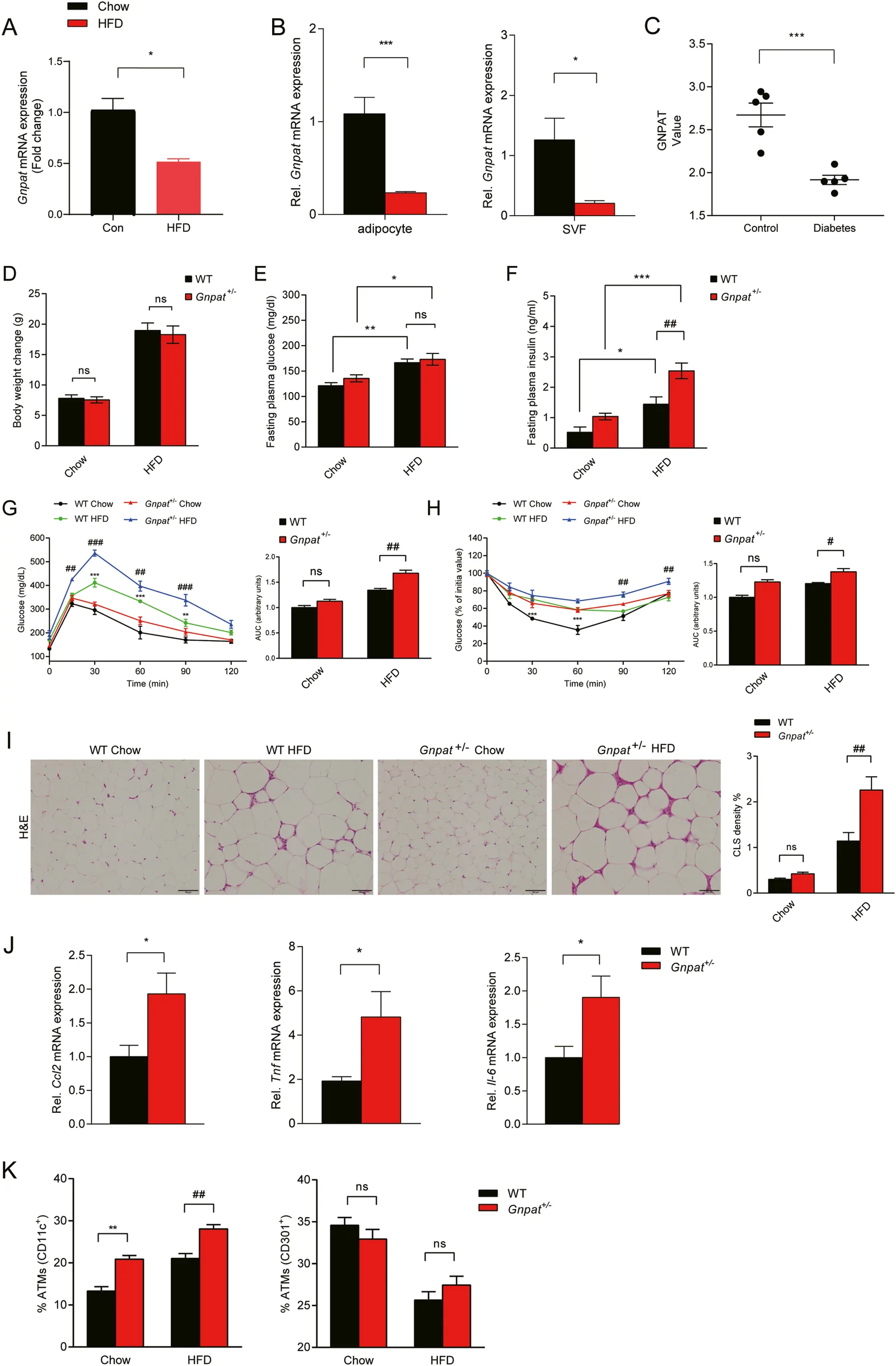
**Haploinsufficiency of GNPAT exacerbates HFD-induced insulin resistance and promotes adipose tissue inflammation and M1 macrophage polarization.** (A, B) *Gnpat* mRNA expression in eWAT, adipocytes, and SVF of HFD-fed mice. (C) mRNA expression of GNPAT in visceral adipose tissue from diabetic versus glucose-tolerant obese women, analyzed by microarray (GSE16415). (D) Body weight, (E) fasting plasma glucose, and (F) fasting plasma insulin levels in WT and *Gnpat*^+/−^ mice after 8 weeks of chow or HFD feeding. (G) GTT and AUC analysis in WT and *Gnpat*^+/−^ mice after 8 weeks of chow or HFD feeding. (H) ITT and AUC analysis. (I) Representative H&E staining of eWAT and quantification of CLS density. Scale bars, 100 μm. (J) Relative mRNA expression of *Ccl2*, *Tnf*, and *Il-6*. (K) Percentage of ATMs stained CD11b^+^F4/80^+^ ATMs among SVF, gated as CD11c^+^ (for M1) or CD301^+^ (for M2). Data are presented as mean ± SEM (n = 4–5). For panels A–I and K, ∗*P* < 0.05, ∗∗*P* < 0.01, and ∗∗∗*P* < 0.005 versus the corresponding chow group; ^#^*P* < 0.05 and ^##^*P* < 0.01 versus the corresponding HFD group; ns, not significant. For panel J, ∗*P* < 0.05 versus WT mice under the same dietary condition.

To investigate whether reduced endogenous plasmalogens contribute to adipose tissue inflammation and insulin resistance, we characterized the metabolic phenotype of mice lacking Gnpat. Since homozygous knockout animals did not survive beyond two weeks, we examined the effects of HFD feeding in *Gnpat*^*+/−*^ mice [18]. Body weight gain in *Gnpat*^*+/−*^ mice fed either a chow diet or an HFD for eight weeks was comparable to that of WT mice (Figure 3D). Similarly, fasting plasma glucose levels were also comparable (Figure 3E). However, HFD-fed *Gnpat*^*+/−*^ mice exhibited significantly higher fasting plasma insulin levels than HFD-fed WT mice (Figure 3F). This was further supported by worsened glucose tolerance in HFD-fed *Gnpat*^*+/−*^ mice according to GTT (Figure 3G) and impaired insulin sensitivity according to the ITT (Figure 3H). Collectively, these findings indicate more severe insulin resistance in mice that are heterozygous for GNPAT.

Histological analysis revealed that HFD-fed *Gnpat*^*+/−*^ mice exhibited significantly higher numbers of macrophages infiltrating adipose tissue compared to HFD-fed WT mice (Figure 3I). Correspondingly, the mRNA levels of proinflammatory cytokines were elevated in the adipose tissue of HFD-fed *Gnpat*^*+/−*^ mice compared with WT mice (Figure 3J). Adipose tissue explants from HFD-fed *Gnpat*^*+/−*^ mice promoted the M1 phenotype of ATMs (CD11b^+^F4/80^+^CD11c^+^) compared to WT mice, whereas the M2 phenotype of ATMs (CD11b^+^F4/80^+^CD301^+^) remained unchanged in HFD-fed *Gnpat*^*+/−*^ mice (Figure 3K). When AG was administered, the proinflammatory mRNA expression and M1 macrophage polarization induced by GNPAT inhibition were reversed; however, the expression of M2-associated markers showed no significant changes (Supplementary Fig. 8). Taken together, these results suggest that partial loss of GNPAT increases susceptibility to HFD-induced adipose tissue inflammation and systemic insulin resistance by promoting M1 macrophage activation.

### 2.5 GNPAT suppresses M1 macrophage activation by inhibiting PKCδ–STAT1 signaling and modulating the balance between plasmalogen and DAG metabolism

Our in vivo studies demonstrated that GNPAT deficiency exacerbated HFD-induced inflammation, while AG supplementation mitigated this response without affecting markers of the anti-inflammatory macrophage phenotype. To validate these findings, we examined the effect of AG treatment on lipopolysaccharide (LPS)-induced activation in murine J774 macrophages. As expected, LPS stimulation strongly induced M1 polarization, evidenced by a significant upregulation of proinflammatory markers, including iNOS, *Ccl2*, and *Tnf* (Supplementary Fig. 9A). Notably, AG treatment combined with LPS markedly attenuated the expression of these M1-associated markers, suggesting that AG suppresses M1 macrophage activation (Supplementary Fig. 9A). Conversely, AG did not significantly affect the expression of M2 macrophage markers, such as *Arg1* and CD206, in LPS-stimulated cells (Supplementary Fig. 9B), indicating that AG attenuates M1 polarization without promoting M2 differentiation under LPS stimulation.

To further investigate the role of GNPAT in macrophage inflammatory responses, we overexpressed GNPAT in J774 macrophages using a lentiviral system (Supplementary Fig. 10A). GNPAT overexpression resulted in a significant reduction in LPS-induced mRNA expression of inflammation-related genes (Figure 4A).

**Figure 4:**
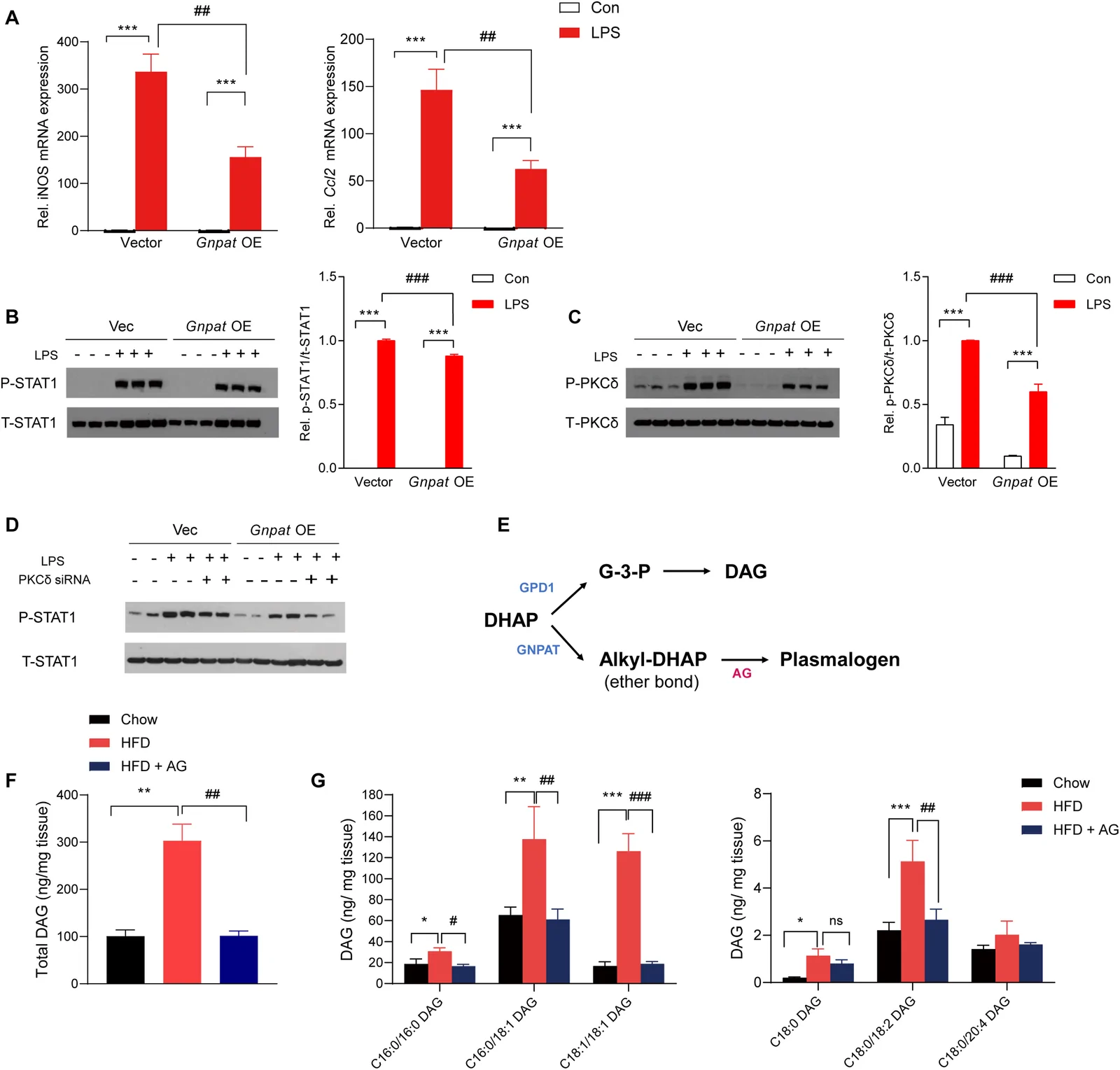
**GNPAT inhibits PKCδ–STAT1 signaling by regulating DAG biosynthesis in macrophages and adipose tissue.** (A) Relative mRNA expression levels of proinflammatory genes in J774 macrophages transduced with a lentiviral control (Vector) or GNPAT overexpression vector (*Gnpat* OE), followed by LPS stimulation. (B) Western blot analysis of phosphorylated and total STAT1 in J774 macrophages with or without GNPAT overexpression after LPS treatment. Quantification of the p-STAT1/t-STAT1 ratio is shown. (C) Representative Western blot showing phosphorylated and total PKCδ in macrophages overexpressing GNPAT, with quantification of the p-PKCδ/t-PKCδ ratio. (D) Macrophages were co-transfected with PKCδ siRNA and GNPAT-expressing vectors, followed by LPS treatment. Representative Western blot analysis of phosphorylated and total STAT1 is shown. (E) Schematic representation of the metabolic pathways leading from DHAP to DAG and plasmalogen. DHAP, dihydroxyacetone phosphate; GPD1, glycerol-3-phosphate dehydrogenase 1; G-3-P, glycerol-3-phosphate; DAG, diacylglycerol; GNPAT, glyceronephosphate O-acyltransferase; Alkyl-DHAP, alkyl-dihydroxyacetone phosphate; AG, alkyl glycerol. (F) Quantification of total DAG in eWAT from chow-fed, HFD-fed, and HFD plus AG-treated mice. (G) Quantification of individual DAG species in eWAT from the indicated groups. Data are presented as mean ± SEM (n = 3–6). For panels A–C, ∗∗∗*P* < 0.005 versus the Control group; ^##^*P* < 0.01 and ^###^*P* < 0.005 versus the Vector group; ns, not significant. For panels F and G, ∗*P* < 0.05, ∗∗*P* < 0.01, and ∗∗∗*P* < 0.005 versus the chow group; ^#^*P* < 0.05, ^##^*P* < 0.01, and ^###^*P* < 0.005 versus the HFD group.

To directly determine whether GNPAT regulates plasmalogen metabolism within macrophages, we performed lipidomic analysis in control and GNPAT-overexpressing J774 macrophages following LPS stimulation. LPS markedly reduced the abundance of the measured PE-plasmalogen species, including 18:0_18:1 PE, 18:0_20:4 PE, and 18:0_22:6 PE. GNPAT overexpression significantly restored all three PE-plasmalogen species under LPS-stimulated conditions (Supplementary Fig. 10B).

In contrast, the measured PC-plasmalogen species were not significantly altered by LPS stimulation alone. However, GNPAT overexpression significantly increased these PC-plasmalogen species in LPS-stimulated macrophages compared with LPS-treated control cells (Supplementary Fig. 10C). These findings provide direct cellular evidence that GNPAT enhances macrophage plasmalogen biosynthesis under inflammatory conditions, while also indicating differential responses of PE- and PC-plasmalogen pools to LPS stimulation.

We then examined the upstream factors contributing to this anti-inflammatory effect. STAT1, a key transcription factor involved in LPS-induced M1 polarization and adipose tissue inflammation, is essential for proinflammatory gene expression and has been implicated in obesity-associated insulin resistance [[34], [35], [36]]. We found that GNPAT overexpression markedly suppressed STAT1 phosphorylation in LPS-stimulated macrophages (Figure 4B), suggesting that GNPAT inhibits M1 macrophage activation by downregulating STAT1 signaling.

To elucidate the upstream signaling mechanisms underlying GNPAT-mediated inhibition of STAT1, we first investigated whether other major inflammatory pathways were affected. Upon LPS stimulation, multiple signaling cascades, including JNK and NF-κB, are typically activated alongside STAT1 to coordinate proinflammatory gene expression [37]. Therefore, we assessed the phosphorylation status of JNK and IκB-α in macrophages overexpressing GNPAT. Notably, phosphorylation of neither JNK nor IκB-α was altered by GNPAT overexpression (Supplementary Fig. 11). Since the JNK and NF-κB pathways remained unaffected, these data suggest that GNPAT exerts its anti-inflammatory effects through a mechanism specifically targeting STAT1. Interestingly, plasmalogens attenuate neuroinflammation by downregulating PKCδ expression in microglia and Alzheimer's disease model brains [38]. Also, PKCδ has been shown to mediate STAT1 serine phosphorylation [39]. PKCδ phosphorylation was markedly increased by LPS treatment, whereas GNPAT overexpression attenuated this effect (Figure 4C). To investigate the role of PKCδ in LPS-induced STAT1 activation, macrophages were transiently transfected with PKCδ siRNA in the context of GNPAT overexpression and subsequently treated with LPS. PKCδ silencing markedly inhibited the induction of STAT1 phosphorylation in response to LPS, demonstrating that PKCδ mediates LPS-induced STAT1 activation in macrophages (Figure 4D). Collectively, these findings demonstrate that GNPAT suppresses STAT1 activation in response to LPS via a PKCδ-dependent mechanism. This novel pathway highlights the role of GNPAT in attenuating adipose tissue inflammation by inhibiting PKCδ activity, thereby reducing STAT1 phosphorylation.

Because Ccl2 was consistently suppressed by AG treatment and GNPAT overexpression in our inflammatory gene analyses and is a key chemokine involved in monocyte/macrophage recruitment and obesity-associated adipose tissue inflammation [40], we selected Ccl2 as a representative inflammatory target for further analysis. To determine whether GNPAT-mediated suppression of STAT1 signaling is functionally linked to Ccl2 transcription, we examined the activity of a Ccl2 promoter reporter containing a STAT1-binding regulatory element. LPS markedly increased *Ccl2* promoter activity in J774 macrophages, whereas GNPAT overexpression significantly attenuated LPS-induced promoter activity (Supplementary Fig. 12). Together with the observed reduction in STAT1 phosphorylation, these findings support a functional link between GNPAT-mediated suppression of STAT1 signaling and reduced Ccl2 transcription.

We next asked whether modulation of macrophage inflammatory activation has functional consequences for neighboring adipocytes. Conditioned medium collected from LPS-stimulated J774 macrophages markedly impaired insulin-stimulated Akt phosphorylation in differentiated 3T3-L1 adipocytes. In contrast, conditioned medium derived from GNPAT-overexpressing macrophages significantly attenuated this impairment (Supplementary Fig. 13). These findings indicate that modulation of macrophage inflammatory activation influences adipocyte insulin sensitivity through a paracrine mechanism, providing a functional link between macrophage GNPAT/plasmalogen metabolism and adipocyte insulin resistance.

Finally, to further elucidate how GNPAT and plasmalogens suppress PKCδ activation, we investigated the ether lipid biosynthesis pathway, emphasizing its interaction with diacylglycerol (DAG) metabolism. Our previous research demonstrated that elevated DAG levels directly contribute to PKCδ activation in the livers of murine models of MASH [41]. Ether lipid and DAG synthesis are metabolically distinct pathways that diverge from a common precursor, dihydroxyacetone phosphate (DHAP). Glycerol-3-phosphate dehydrogenase 1 (GPD1) reduces DHAP to glycerol-3-phosphate (G3P), which is subsequently converted into DAG and triacylglycerol via the glycerolipid pathway. In contrast, GNPAT catalyzes the acylation of DHAP, directing it toward ether lipid and plasmalogen synthesis [42]. Therefore, the relative activities of GPD1 and GNPAT critically determine the metabolic fate of DHAP [43], modulating the balance between glycerolipid and ether lipid biosynthesis (Figure 4E). Consistent with previous findings, HFD feeding elevated DAG levels [44], whereas AG treatment significantly reduced them (Figure 4F,G). Together, these findings suggest that enhanced GNPAT-dependent ether lipid metabolism may limit DAG accumulation and subsequent PKCδ–STAT1 activation, thereby attenuating macrophage inflammatory responses under metabolic stress.

## 3 Discussion

In this study, we demonstrate that GNPAT-mediated plasmalogen biosynthesis plays a crucial role in regulating adipose tissue inflammation and insulin resistance under metabolic stress conditions. Enhancing plasmalogen levels through AG supplementation improved insulin sensitivity and reduced M1 macrophage–driven inflammation in HFD-fed mice, whereas GNPAT deficiency was associated with increased insulin resistance and exacerbated adipose tissue inflammation. Notably, AG administration initiated after the establishment of diet-induced insulin resistance also improved glucose homeostasis, indicating therapeutic efficacy. This effect appears to be mediated by suppression of the PKCδ–STAT1 signaling pathway in macrophages, suggesting that ether lipid metabolism helps limit proinflammatory responses in adipose tissue.

Plasmalogens, a distinctive class of phospholipids, are synthesized in peroxisomes and the endoplasmic reticulum and are widely distributed across mammalian tissues [29], including adipocyte membranes. Deficiency of the peroxisomal protein PEX7 results in plasmalogen depletion and pathological changes in white adipose tissue, both of which can be ameliorated by AG supplementation [23], highlighting their role in adipose biology. In brown adipocytes, plasmalogen supplementation enhances mitochondrial function and restores cold-induced thermogenesis [45]. In our study, although plasmalogen supplementation did not affect body weight, it significantly improved insulin sensitivity by suppressing adipose inflammation and M1 macrophage activation. These findings suggest that plasmalogens play an important role in maintaining immune-metabolic homeostasis in adipose tissue and mitigating obesity-related insulin resistance.

Beyond its effects on adipose tissue inflammation, our additional experiments suggest that AG also improves insulin responsiveness in peripheral metabolic tissues. AG enhanced insulin signaling in skeletal muscle both in vivo and in insulin-resistant C2C12 myotubes, accompanied by improved GLUT4 trafficking. Together with similar improvements in insulin signaling in adipocytes, these findings suggest that the metabolic benefits of AG extend beyond its anti-inflammatory effects and involve enhanced insulin responsiveness across multiple peripheral tissues.

Macrophages in lean adipose tissue predominantly exhibit an M2-like, anti-inflammatory phenotype, whereas obesity induces a shift toward a proinflammatory M1-like phenotype, contributing to chronic inflammation and insulin resistance. Here, we demonstrate that AG treatment reduces both macrophage infiltration and the number of M1-like macrophages in the epididymal adipose tissue of HFD-fed mice. In contrast, *Gnpat*^+^/^−^ mice exhibit exacerbated adipose tissue inflammation, characterized by a marked increase in M1-like macrophages, with no significant change in M2-like populations. Consistent with these in vivo findings, AG selectively suppresses LPS-induced M1 polarization in cultured macrophages without altering the expression of M2 markers. Together, these results suggest that the anti-inflammatory effects of plasmalogens are primarily mediated by inhibition of M1 macrophage activation rather than by promotion of M2 phenotypes.

Although previous studies have highlighted the role of iNKT cells in mediating anti-inflammatory responses in adipose tissue via CD1d-mediated lipid antigen presentation [[25], [26], [27]], our findings suggest that AG operates via an alternative pathway. While AG supplementation partially restored iNKT cell populations in HFD-fed mice, its anti-inflammatory and insulin-sensitizing effects remained evident in *Cd1d*^*−/−*^ mice lacking iNKT cells [28]. Instead, AG directly modulated macrophage polarization by suppressing the pro-inflammatory M1 phenotype through GNPAT-driven plasmalogen biosynthesis and suppression of PKCδ–STAT1 signaling in macrophages. These results indicate that AG may exert anti-inflammatory effects independently of iNKT cell activation.

Plasmalogens, which are enriched with AA and DHA, play a pivotal role in inflammatory responses. They constitute approximately 15% of cellular lipids in circulating monocytes and are essential for phagocyte function and protection against oxidative stress [46,47]. In macrophages and neutrophils, up to 40% of PE-plasmalogens contain AA, providing a reservoir for lipid mediators involved in inflammatory responses [48], whereas DHA-containing plasmalogens may contribute to the generation of pro-resolving lipid mediators, including resolvins and protectins [49]. In our study, HFD feeding altered adipose tissue plasmalogen composition, characterized by an increase in AA-enriched species and a decrease in DHA-containing species, whereas AG supplementation partially reversed this pattern by restoring several DHA-containing plasmalogen species. Consistent with these tissue-level findings, lipidomic analysis of macrophages demonstrated that GNPAT overexpression increased both PE- and PC-plasmalogens under inflammatory conditions, including DHA-containing plasmalogen species. Plasmalogens, particularly ethanolamine plasmalogens, are dynamically involved in membrane lipid remodeling during macrophage inflammatory activation and serve as an important reservoir for polyunsaturated fatty acids [50,51]. Together, these findings indicate that the anti-inflammatory effects of AG and GNPAT involve both restoration of the overall plasmalogen pool and remodeling of specific PUFA-containing species. In particular, restoration of DHA-containing plasmalogens may further promote an anti-inflammatory and pro-resolving lipid environment, thereby contributing to the suppression of macrophage inflammatory activation.

We investigated the molecular mechanisms underlying the anti-inflammatory effects of plasmalogens, with a particular focus on the role of GNPAT in modulating lipid-mediated signaling pathways. Our results demonstrate that GNPAT overexpression in macrophages suppresses LPS-induced activation of PKCδ, leading to reduced STAT1 phosphorylation and downstream inflammatory gene expression. This finding aligns with previous studies indicating that PKCδ is a critical upstream kinase responsible for STAT1 serine 727 phosphorylation during inflammatory responses [52]. Notably, GNPAT overexpression did not significantly alter JNK or NF-κB signaling, suggesting a relatively selective effect on the PKCδ–STAT1 pathway. Our additional promoter analysis further supports the functional relevance of STAT1 signaling to inflammatory transcription. CCL2 plays an important role in monocyte recruitment and macrophage accumulation in adipose tissue and contributes to obesity-associated inflammation and insulin resistance [40]. Using a *Ccl2* promoter reporter containing a STAT-binding regulatory element, we found that GNPAT overexpression attenuated LPS-induced Ccl2 promoter activity. Together with the observed suppression of PKCδ and STAT1 activation, these findings support a functional link between GNPAT-mediated regulation of the PKCδ–STAT1 pathway and Ccl2 transcription, providing a transcriptional mechanism through which GNPAT may suppress macrophage inflammatory activation.

Previously, we reported that DAG produced through sphingomyelin synthase 1 activity can activate PKCδ and contribute to pyroptosis in hepatocytes in a murine model of MASH [41]. In the current study, we also observed an accumulation of DAG—a key activator of PKCδ—following HFD administration. Conversely, AG treatment led to a reduction in DAG levels in eWAT. Because ether lipid and glycerolipid biosynthesis share DHAP as a common metabolic precursor, alterations in GNPAT-dependent ether lipid synthesis may influence the partitioning of this precursor between these pathways [43,53]. Thus, our findings suggest that GNPAT serves as a key metabolic regulator by diverting DHAP from DAG biosynthesis toward ether lipid synthesis. By limiting DAG availability, GNPAT indirectly suppresses PKCδ activation and its downstream inflammatory cascade. Further studies are warranted to determine whether plasmalogens themselves directly interfere with PKCδ function or whether their effects are entirely mediated by substrate flux and lipid balance.

Although the systemic *Gnpat* haploinsufficient model does not allow definitive assignment of the in vivo phenotype to a specific cell population, our findings point to macrophages as a principal cellular contributor. The effects of GNPAT on inflammatory signaling were consistently observed in macrophages, and our conditioned-medium experiments further demonstrated that macrophage GNPAT status functionally influences insulin responsiveness in neighboring adipocytes. Conditioned medium from LPS-stimulated macrophages impaired insulin-stimulated AKT phosphorylation in differentiated 3T3-L1 adipocytes, whereas this impairment was attenuated when GNPAT was overexpressed in macrophages. These findings place macrophage GNPAT upstream of adipocyte insulin resistance through a paracrine mechanism and support macrophages as a major driver of the adipose inflammatory and metabolic phenotype observed in vivo. Nevertheless, adipocyte-intrinsic and other cellular contributions cannot be excluded, and macrophage- and adipocyte-specific *Gnpat-*deficient models will be required to define their relative contributions in vivo.

The present in vivo studies were conducted in male mice, and potential sex-dependent differences should also be considered. Given the known sex-specific differences in adipose inflammation and lipid metabolism, whether the GNPAT–plasmalogen axis exerts similar metabolic effects in females remains to be determined.

Importantly, species differences in peroxisome biology should be considered when interpreting translational relevance. As discussed by Islinger et al. [54], peroxisome abundance and functional characteristics vary substantially across species and tissues. Nevertheless, human studies have reported alterations in peroxisomal lipid metabolism and reduced plasmalogen levels in obesity and type 2 diabetes [55,56]. Furthermore, clinical intervention studies using oral AG or plasmalogen precursor supplementation have demonstrated increased circulating plasmalogen levels and improvements in metabolic and inflammatory parameters in humans [57,58]. Together with the reduced GNPAT expression observed in human diabetic adipose tissue in the present study, these findings support further investigation of the GNPAT–plasmalogen pathway in human metabolic disease. Given the involvement of PKCδ–STAT1 signaling in metabolic inflammation, studies in human adipocytes and macrophages, followed by appropriately designed clinical studies, will be important to determine the translational potential of targeting this pathway.

In conclusion, our findings suggest that modulating plasmalogen biosynthesis, particularly through GNPAT activation, may represent a novel strategy for mitigating adipose tissue inflammation and improving insulin sensitivity. The ability of AG to restore ether lipid balance and suppress PKCδ–STAT1 signaling highlights a potential metabolic–inflammatory interface that can be therapeutically targeted. Notably, the reduced expression of GNPAT in human diabetic adipose tissue underscores the translational relevance of this pathway. Further studies are required to evaluate the therapeutic potential of plasmalogen-enhancing agents or GNPAT-targeted strategies in obesity-related disorders.

## 4 Materials and methods

### 4.1 Mice and diets

Eight-week-old C57BL/6 N mice (Central Laboratory Animal Inc., Seoul, Korea) were housed at an ambient temperature (22 ± 1 °C) under a 12 h:12 h light–dark cycle and had free access to food and water. *Gnpat* knockout mice were generated using the TALEN (transcription activator-like effector nuclease) method, as previously described [18]. As *Gnpat*^−/−^ mice did not survive beyond two weeks, *Gnpat*^+/−^ mice were used for experiments. *Cd1d* knockout mice (#017294) with a C57BL/6 J genetic background were purchased from the Jackson Laboratory (Bar Harbor, ME, USA).

The mice were divided into groups and fed a chow diet (ND; 12% of energy from fat), HFD (60% of energy from fat; Research Diets, Inc., New Brunswick, NJ, USA), or HFD supplemented with 1-O-octadecyl-rac-glycerol (a commercial form of AG; also called batyl alcohol; Sigma–Aldrich, Saint Louis, MO, USA) at a dose of 10 mg/kg per day for 8 weeks. For the treatment model, mice were fed an HFD for 4 weeks to induce obesity, followed by HFD supplemented with AG (10 mg/kg/day) for an additional 4 weeks. After the 8-week experimental period, the mice were fasted for 5 h in the morning and then sacrificed.

All animal experiments were approved by the Institutional Animal Care and Use Committee of the Asan Institute for Life Sciences (approval number 2019-12-301).

### 4.2 Glucose tolerance test and insulin tolerance test

For the intraperitoneal GTT, mice were fasted overnight for 12 h and then injected intraperitoneally with glucose (1 g/kg body weight). For the intraperitoneal ITT, mice were fasted for 5 h in the morning and injected intraperitoneally with human insulin (0.5 U/kg body weight). Blood samples were collected from the tail vein at various time points.

### 4.3 GSIS test

The GSIS test was performed as previously described [59]. Briefly, mice were fasted overnight. After fasting, glucose (1.0 g/kg of body weight) was administered via intraperitoneal injection. Blood samples were collected from the tail vein at 0, 5, 15, 30, and 60 min post-injection. Serum insulin levels were measured by ELISA (#292–89401, FUJIFILM Wako Pure Chemical Corporation). The AUC for insulin secretion was calculated.

### 4.4 Acute insulin stimulation

To evaluate acute insulin signaling, mice were fasted for 6 h and then injected intraperitoneally with insulin (0.75 U/kg body weight) or an equivalent volume of PBS. After 10 min, the mice were sacrificed, and the gastrocnemius muscles were rapidly excised, snap-frozen in liquid nitrogen, and stored at −80 °C until further analysis.

### 4.5 Plasma biochemical assays

Plasma glucose levels were determined using a glucose analyzer (YSI 2300, Yellow Springs Instruments). Plasma insulin levels were measured using radioimmunoassay kits (Linco Research).

### 4.6 Histological analysis

eWAT was rapidly excised from each mouse. The tissues were immediately fixed in 10% formalin, dehydrated, embedded in paraffin, and sectioned for staining. For morphological analysis, sections were stained with hematoxylin and eosin (H&E). For immunohistochemistry, antigen retrieval was performed with pre-heated citrate buffer (pH 6.0), followed by incubation with peroxidase blocking solution (#S2023, Dako) for 30 min. Tissue slides were then blocked with blocking solution for 30 min. After blocking, slides were incubated with a primary antibody against F4/80 (dilution 1:100; #ab11101, Abcam) in blocking solution at RT for 1 h. Subsequently, a biotinylated secondary antibody (#PK-4001, Vector Laboratories) was applied for 30 min, followed by incubation with Vectastain ABC reagent for 30 min. Diaminobenzidine tetrahydrochloride was applied to the slides and immediately washed before mounting. Representative images were captured using a microscope (#BX53, Olympus). The density of crown-like structures (CLS) and the F4/80-positive area were quantified from H&E− and F4/80-stained images, respectively, using ImageJ software.

### 4.7 Targeted lipidomics analysis of plasmalogens and DAG

Lipid extraction and quantification were performed as part of a targeted lipidomics analysis using LC-MS/MS, as described below. For tissue lipidomics, mouse eWAT (40–50 mg) was homogenized, and an internal standard solution (50 μL of 10 μM 1,3–19:0 D5 DAG) was added prior to lipid extraction. For macrophage lipidomics, control-vector and GNPAT-overexpressing J774 macrophages were cultured under identical conditions and stimulated with LPS (5 ng/mL) for 16 h. Following treatment, cells were washed with ice-cold phosphate-buffered saline, harvested, and subjected to lipid extraction. Lipids were extracted using the Folch method [60]. The organic phase was dried using a vacuum centrifuge and stored at −20 °C until analysis. The dried extract was reconstituted in methanol and subjected to LC-MS/MS analysis.

Plasmalogen levels were determined using a 1290 HPLC system (Agilent) coupled with a QTRAP 5500 mass spectrometer (AB Sciex), as previously described [18]. For DAG quantification, a 1290 HPLC system (Agilent) paired with a TRIPLE QUAD 6500 mass spectrometer (AB Sciex) was employed. Chromatographic separation was conducted on a Zorbax Eclipse C18 column (50 × 2.1 mm) using mobile phase A (10 mM ammonium acetate in MeOH/isopropanol/H_2_O, 900/50/50) and mobile phase B (10 mM ammonium acetate in MeOH/isopropanol/H_2_O, 940/50/10). The liquid chromatography run was performed isocratically at 95% mobile phase B with a flow rate of 400 μL/min for 20 min. Multiple reaction monitoring (MRM) in positive ion mode was utilized, and quantification was based on extracted ion chromatograms for each lipid species. The calibration range for each lipid was 1–10,000 nM (r^2^ ≥ 0.99). Data analysis was conducted using Analyst 1.6.2 software. All standard metabolites and internal standards were purchased from Avanti Polar Lipids.

### 4.8 Isolation of adipocytes and SVF from adipose tissue

For the isolation of primary adipocytes, eWAT was minced and digested for 30 min at 37 °C with type II collagenase (1 mg/mL; #C6885, Sigma–Aldrich). The cell suspensions were filtered through a 100 μm mesh and centrifuged at 500×*g* for 10 min to separate the mature adipocyte fraction (floating layer) from the SVF (pellet). The SVF pellet was resuspended in red blood cell lysis buffer (#R7757, Sigma–Aldrich) and incubated at room temperature for 5 min to lyse erythrocytes. After lysis, the cells were centrifuged again at 500×*g* for 5 min. The resulting SVF pellet was washed with PBS and resuspended in an appropriate buffer for further analysis, as previously described [61].

### 4.9 Flow cytometry analysis

SVFs were isolated from adipose tissue and stained with fluorochrome-conjugated antibodies for flow cytometry analysis. To identify ATMs, cells were incubated with antibodies against CD45 (#45-0454-82, Invitrogen), F4/80 (#12-4801-82, Invitrogen), CD11b (#47-0112-82, Invitrogen), CD301 (#MCA2392A647, Bio-Rad), and CD11c (#25-0114-82, Invitrogen). M1 (CD11c^+^) and M2 (CD301^+^) ATMs were identified using the gating strategy described in Supplementary Fig. 6A.

For iNKT cell analysis, SVFs were stained with TCRβ antibody (#11-5961-82, Invitrogen), mCD1d/PBS-57 tetramer (#557599, BD Biosciences), and PE-conjugated rat anti-mouse IgG1 antibody (#550083, BD Biosciences) according to the manufacturer's instructions. After staining, all cells were washed with FACS buffer (PBS containing 2% FBS) and analyzed using a BD FACS Canto II (BD Biosciences) (Supplementary Fig. 5A). Data were processed using FlowJo software.

### 4.10 Real-time PCR analysis

Total RNA was isolated using TRIzol reagent (#15596018, Invitrogen) and reverse-transcribed into cDNA using the High-Capacity cDNA Reverse Transcription Kit (#K1691, Thermo Fisher Scientific). Quantitative reverse transcription polymerase chain reaction (qRT-PCR) was performed using SYBR Green dye (#RT501UM, Enzynomics) according to the manufacturer's instructions. Primers were designed based on nucleotide sequences obtained from the GenBank database (Supplementary Table 1). Each reaction was run in triplicate to determine the threshold cycle (Ct) for each mRNA, and relative cDNA expression levels were calculated using the 2^−ΔΔCt method, with Tbp serving as the internal control.

### 4.11 GNPAT overexpression

To generate a vector for overexpressing *Gnpat*, mouse GNPAT cDNA fragments were amplified by PCR (GNPAT ORF-F: CCG GAA TTC ACC ATG TTG TCT GCC AGG AC, GNPAT ORF-R: CGC GGA TCC TTA TGT GTC GTT TAC CAG CC). The PCR products were subcloned into the pCDH-MCS lentiviral vector (CD513B-1, System Biosciences). Each plasmid was transfected into Lenti-X 293 T cells (#632180, Clontech) along with packaging plasmids (pCMV-VSV-G, pMDLg/PRRE, and pRSV-Rev; #8454, #12251, #12253, Addgene) using Lipofectamine 3000 (#L3000-015, Invitrogen). To generate stable transfectants, J774 cells were infected with either control or GNPAT lentiviruses. After 96 h of infection, the cells were cultured in RPMI 1640 medium containing 2 μg/mL puromycin.

### 4.12 Cell culture and treatment of J774 macrophages

J774 cells were maintained in Dulbecco's Modified Eagle's Medium (DMEM) supplemented with 10% fetal bovine serum (FBS) under a humidified atmosphere containing 5% CO_2_ at 37 °C. The cells were seeded in 6-well plates at a density of 2 × 10^5^ cells per well and incubated for 24 h. The culture medium was then replaced with serum-free RPMI 1640 and incubated for 2 h. Subsequently, the cells were preincubated with 50 μM AG for 1 h prior to LPS treatment. The cells were then stimulated with 5 ng/mL LPS (L2880, O55:B5; Sigma–Aldrich) in RPMI 1640 medium containing 1% FBS for 16 h. For Western blot analysis to detect phosphorylated IκBα, JNK, STAT1, and PKCδ, the cells were stimulated with LPS for 6 h.

### 4.13 Differentiation of 3T3-L1 adipocytes and assessment of insulin signaling

To evaluate the insulin signaling pathway, the mouse pre-adipocyte 3T3-L1 cell line was cultured in DMEM supplemented with 10% FBS. Upon reaching 70% confluence, differentiation was induced by culturing the cells in DMEM containing 10% FBS, supplemented with 1 μM dexamethasone (D2915, Sigma–Aldrich), 0.5 mM 3-isobutyl-1-methylxanthine (IBMX) (I5879, Sigma–Aldrich), and 10 μg/mL insulin (I9278, Sigma–Aldrich). On day 3 of differentiation, the medium was replaced with DMEM containing 10% FBS and 10 μg/mL insulin. On day 5 of differentiation, the culture medium was refreshed with DMEM containing 10% FBS. Differentiated 3T3-L1 cells were serum- and insulin-starved for 6 h. The cells were then preincubated with 50 μM AG for 1 h prior to treatment with 200 μM palmitate or 2% BSA. After 24 h of palmitate treatment, the cells were stimulated with 10 nM insulin for 3 min to assess insulin signaling.

### 4.14 Differentiation of C2C12 myotubes and assessment of insulin signaling and GLUT4 translocation

C2C12 mouse skeletal muscle cells were maintained in DMEM supplemented with 10% FBS and 1% penicillin–streptomycin at 37 °C in a humidified atmosphere containing 5% CO_2_. When the cells reached approximately 90% confluence, myogenic differentiation was induced by replacing the growth medium with DMEM containing 2% horse serum. The differentiation medium was replaced every 48 h, and fully differentiated myotubes were used for experiments after 6 days. To induce insulin resistance, differentiated C2C12 myotubes were pre-treated with 10 μM AG for 48 h, followed by exposure to 500 μM PA in serum-free medium for 24 h with or without AG. Finally, the cells were stimulated with 100 nM insulin for 30 min under serum-starved conditions. Whole-cell lysates were collected and subjected to immunoblot analysis using antibodies against phosphorylated AKT and total AKT.

To assess insulin-stimulated GLUT4 recruitment to the plasma membrane, plasma membrane fractions were isolated from C2C12 myotubes using (#89842, Thermo Fisher Scientific) according to the manufacturer's instructions. Equal amounts of protein from the plasma membrane fractions were subjected to immunoblot analysis for GLUT4 (#2213, Cell Signaling). Na^+^/K^+^-ATPase (#3010, Cell Signaling) was used as a plasma membrane marker to verify membrane fraction enrichment. Plasma membrane GLUT4 abundance was quantified by densitometric analysis and normalized to Na^+^/K^+^-ATPase.

### 4.15 Preparation of macrophage-conditioned medium and treatment of 3T3-L1 adipocytes

To investigate the paracrine effects of macrophage activation on adipocyte insulin signaling, control-vector and GNPAT-overexpressing J774 macrophages were stimulated with 1 ng/mL LPS for 16 h. Following treatment, culture supernatants were collected and filtered to remove cellular debris. The resulting macrophage-conditioned medium was then transferred to fully differentiated 3T3-L1 adipocytes. After incubation with macrophage-conditioned medium for 24 h, 3T3-L1 adipocytes were serum-starved for 6 h and subsequently stimulated with 10 nM insulin for 3 min. Cells were then harvested, and insulin signaling was evaluated by immunoblot analysis of phosphorylated and total AKT.

### 4.16 PKCδ siRNA transfection

J774 cells were seeded in 6-well plates at a density of 1 × 10^5^ cells per well and grown to approximately 50% confluence. PKCδ siRNAs were purchased from Bioneer. The sequences (5′ → 3′) of the siRNAs were as follows: PKCδ sense: CUU UAA GCC CAA AGU GAA Att; PKCδ antisense: UUU CAC UUU GGG CUU AAA Gtt. Transfections were performed using Lipofectamine 3000 for 48 h at a final siRNA concentration of 50 nM. Subsequently, cells were treated with 5 ng/mL LPS in RPMI 1640 medium containing 1% FBS for 6 h.

### 4.17 Western blot analysis

For Western blot analysis, J774 cells were lysed, and equal amounts of protein were electrophoresed on 10% SDS-PAGE gels. The separated protein bands were transferred to a nitrocellulose membrane for 90 min. After blocking with 5% non-fat milk for 1 h, the membranes were incubated overnight at 4 °C with the following primary antibodies: phospho-STAT1 Ser727, STAT1 (#14994, Cell Signaling), phospho-PKCδ Tyr311 (#2055, Cell Signaling), PKCδ (#2058, Cell Signaling), phospho-AKT Ser473 (#9271, Cell Signaling), AKT (#9272, Cell Signaling). The membranes were then rinsed with TBST buffer and incubated with a secondary antibody (#406401, BioLegend) for 1 h at room temperature. The blots were developed using Western Blot Substrate (#BWD0100, Biomax) and exposed to X-ray film. Equal protein loading was confirmed using a GAPDH antibody (#2118, Cell Signaling).

### 4.18 *Ccl2* promoter luciferase reporter assay

To assess *Ccl2* transcriptional activity, a mouse *Ccl2* promoter fragment (−458 to +1 bp) containing a STAT1-binding regulatory element was cloned into a Gaussia luciferase reporter vector. J774 cells were seeded in 24-well culture plates and subsequently co-transduced with *Ccl2* promoter lentivirus (MOI 20) and GNPAT overexpression lentivirus (MOI 5–25) in the presence of 8 μg/mL polybrene (H9268, Sigma–Aldrich) for 6 h. Following transduction, cells were treated with 5 ng/mL LPS for 24 h. Culture media were then collected, and Gaussia luciferase activity was measured using the Secrete-Pair™ Gaussia Luciferase Dual Luminescence Assay Kit (LF033, GeneCopoeia) and a VICTOR *X*2 luminometer (PerkinElmer), according to the manufacturers’ instructions.

### 4.19 Bioinformatic analysis of human adipose tissue transcriptomic datasets

Publicly available human adipose tissue transcriptomic datasets were obtained from the NCBI GEO database to evaluate the clinical relevance of GNPAT expression in metabolic dysfunction. Two independent datasets, GSE16415 and GSE20950, were analyzed. GSE16415 was used to compare GNPAT expression in visceral adipose tissue between women with type 2 diabetes and age- and BMI-matched women without diabetes. The dataset included omental adipose tissue samples from five women with type 2 diabetes and five control women undergoing cholecystectomy. All participants were older than 55 years and had a BMI >30 kg/m^2^. GSE20950 was additionally analyzed to examine GNPAT expression in relation to insulin resistance. This dataset included adipose tissue samples from 20 individuals (14 women; mean age, 42 years), who were classified as insulin-sensitive (HOMA2-IR <2.4; n = 10) or insulin-resistant (HOMA2-IR ≥2.4; n = 10).

### 4.20 Statistical analysis

Data are expressed as mean ± standard error of the mean (SEM). An unpaired two-tailed Student's *t*-test was used for comparisons between two groups. One-way ANOVA was used to compare multiple groups, followed by Bonferroni correction for post hoc multiple comparisons. All statistical tests were two-sided, and sample sizes were determined based on previous experiments using similar methodologies. In all experiments, replicates were biological replicates. Statistical analyses and graphing were performed using GraphPad Prism 10 (GraphPad Software).

## CRediT authorship contribution statement

**Ji-Young Yun:** Funding acquisition, Data curation. **Myoung Seok Ko:** Writing – original draft, Investigation, Formal analysis, Data curation. **Chung Hwan Hong:** Writing – review & editing, Investigation, Formal analysis, Data curation. **Ji Eun Yoon:** Investigation, Formal analysis, Data curation. **Jaechan Leem:** Investigation, Formal analysis, Data curation. **Mi-Ok Kim:** Data curation. **Su Jung Kim:** Data curation. **Hyun Ju Yoo:** Formal analysis, Data curation. **Yun-kyung Cho:** Investigation, Formal analysis. **In-Jeoung Baek:** Resources, Data curation. **Young-Bum Kim:** Writing – review & editing, Conceptualization. **Ki-Up Lee:** Writing – review & editing, Writing – original draft, Investigation, Funding acquisition, Conceptualization. **Eun Hee Koh:** Writing – review & editing, Supervision, Investigation, Funding acquisition, Formal analysis, Conceptualization.

## Ethics declaration

This study was conducted in accordance with the ARRIVE (Animal Research: Reporting of In Vivo Experiments) guidelines. This study was approved by the Institutional Animal Care and Use Committee of the Asan Institute for Life Sciences. (Approval No. 2019-12-301)

## Declaration of competing interest

No potential conflicts of interest relevant to this article were reported.

## Data Availability

Data will be made available on request.
