# Supplementary material for: GNPAT-driven plasmalogen biosynthesis regulates macrophage polarization and protects against obesity-induced inflammation by suppressing PKCδ–STAT1 signaling
Source: Mol Metab. 2026 Sep 18;113:102440. doi: 10.1016/j.molmet.2026.102440 (PMC13654497; doi:10.1016/j.molmet.2026.102440)
Supplement: Supplementary file 1 [file mmc1.docx]

**SUPPLEMENTARY DATA**

**Supplementary Figure 1: AG does not alter glucose homeostasis under basal conditions.** (A) GTT and (B) ITT in mice fed a standard chow diet with or without AG supplementation. Data are presented as mean ± SEM (n=5).

**Supplementary Figure 2: Therapeutic administration of AG ameliorates established diet-induced insulin resistance and adipose tissue inflammation.**

(A) Schematic diagram of the therapeutic AG administration protocol (top). Mice were fed either a chow or an HFD for 8 weeks, and AG was administered to the HFD-fed mice from week 4 to week 8. GTT and ITT results, along with their corresponding AUC analyses, are shown. (B) Relative mRNA expression levels of F4/80, CD11c, *Tnf*, *Il-6*, and *Ccl2* in eWAT. Data are presented as mean ± SEM (n=5). ^**^*P* < 0.01, and ^***^*P* < 0.005 versus the chow group. ^#^*P* < 0.05 and ^###^*P* < 0.005 versus the HFD group. ns, not significant.

**Supplementary Figure 3:** **AG restores insulin signaling in skeletal muscle and promotes GLUT4 plasma membrane translocation in insulin-resistant myotubes.**

(A) Representative western blot analysis of phosphorylated AKT (P-AKT), total AKT (T-AKT), and GAPDH, along with quantification of the pAKT/T-AKT ratio in the gastrocnemius muscle of mice fed a chow, HFD, or HFD+AG diet. Mice were intraperitoneally injected with insulin (0.75 U/kg) or vehicle for 10 min prior to tissue collection. GAPDH was used as an internal loading control (n=5). (B) Representative Western blot analysis and quantification of P-AKT and T-AKT in differentiated C2C12 myotubes under control and insulin-resistant (PA-treated) conditions with or without AG treatment, followed by insulin stimulation. GAPDH was used as an internal loading control (n=3). (C) Representative Western blot analysis and quantification of GLUT4 translocation in C2C12 myotubes under the indicated experimental conditions. Na,K-ATPase was used as the loading control for the plasma membrane (PM) fraction. PM-GLUT4 levels were normalized to Na,K-ATPase, and the relative ratio is presented. Total-GLUT4 is shown as a control for total cellular GLUT4 expression (n=3). Data are presented as mean ± SEM. For panel A, ^***^*P* < 0.005 versus the chow-control group; ^###^*P* < 0.005 versus the chow-insulin group; ^$$$^*P* < 0.005 versus the HFD-insulin group. For panel B, ^***^*P* < 0.005 versus the untreated control group; ^##^*P* < 0.01 versus the insulin-stimulated group; ^$$^*P* < 0.01 versus the PA + insulin group. For panel C, ^**^*P* < 0.01 versus the untreated control group; ^##^*P* < 0.01 versus the insulin-stimulated group; ^$^*P* < 0.05 versus the PA + insulin group.

**Supplementary Figure 4:** **AG improves insulin signaling in 3T3-L1 adipocytes but does not affect the expression of lipid metabolism genes in primary adipocytes.**

(A) Representative Western blot analysis of pAKT, AKT, and GAPDH, along with quantification of the pAKT/AKT ratio in differentiated 3T3-L1 adipocytes. Following a 6-hour serum starvation, cells were treated with 200 μM PA in the presence or absence of AG for 24 hours, and subsequently stimulated with 10 nM insulin for 3 min. GAPDH was used as a loading control. (B) Relative mRNA expression of genes related to lipogenesis (*Fasn, Acly, Srebp1c*), fatty acid oxidation (*Cpt1a*), and lipolysis (*Atgl, Hsl*) in the adipocyte fraction isolated from the adipose tissue of mice fed a chow, HFD, or HFD+AG diet. Data are presented as mean ± SEM (n=5). For panel A, ^***^*P* < 0.005 versus control; ^###^*P* < 0.005 versus insulin-stimulated cells; ^$$$^*P* < 0.005 versus PA-treated insulin-stimulated cells. For panel B, ^*^*P* < 0.05, ^**^*P* < 0.01, and ^***^*P* < 0.005 versus the chow group; ns, not significant versus the HFD group.

**Supplementary Figure 5: Improvement of insulin resistance by AG is not mediated through the activation of iNKT cells.**

(A) Single-cell suspensions from epididymal fat were stained with anti-TCRβ and CD1d tetramer, then analyzed by FACS. (B) Bar graphs depict the mean ± SEM of percentages (left) and total numbers (right) of iNKT cells. (C) GTT and AUC analysis in *Cd1d*^-/-^ mice. (D) ITT and AUC analysis in *Cd1d*^-/-^ mice. Data are presented as mean ± SEM (n = 4). ^*^*P* < 0.05, ^**^*P* < 0.01, and ^***^*P* < 0.005 versus the Chow group. ^#^*P* < 0.05 and ^##^*P* < 0.01 versus the HFD group.

**Supplementary Figure 6: Gating strategy for ATMs in eWAT and expression of inflammation-related genes in iWAT.**

(A) Gating strategy used to identify and characterize ATMs in eWAT according to the flow cytometry protocol. Cell aggregates and dead cells (DAPI^+^) were excluded, and adipose tissue leukocytes (CD45^+^) were selected. From the CD45^+^ population, ATMs were identified as F4/80^+^ CD11b^+^ cells and further subtyped into M1 (CD11c^+^) and M2 (CD301^+^) macrophages. (B) Relative mRNA expression of a macrophage marker (F4/80) and pro-inflammatory genes (*Tnf, Ccl2*) in the iWAT of mice fed a chow, HFD, or HFD+AG diet. Data are presented as mean ± SEM (n=5). ^*^*P* < 0.05, ^**^*P* < 0.01, and ^***^*P* < 0.005 versus chow group; ns, not significant versus HFD group.

**Supplementary Figure 7. *GNPAT* and *CCL2* expression in human omental adipose tissue according to insulin sensitivity.**

(A) *GNPAT* mRNA expression in omental adipose tissue from insulin-sensitive and insulin-resistant individuals. (B) *CCL2* mRNA expression in omental adipose tissue from insulin-sensitive and insulin-resistant individuals. Gene expression data were obtained from the publicly available GSE20950 dataset. Data are presented as mean ± SEM (n = 10 per group). ^*^*P* < 0.05 and ^**^*P* < 0.01, as indicated.

**Supplementary Figure 8: AG treatment reverses the pro-inflammatory phenotype in the adipose tissue of HFD-fed Gnpat⁺/⁻ mice.**

(A) mRNA expression levels of pro-inflammatory cytokines were significantly elevated in the adipose tissue of HFD-fed *Gnpat*⁺/⁻ mice compared to WT controls and were markedly reduced following AG administration. (B) Flow cytometric analysis of ATMs showed that the proportion of M1-like ATMs (CD11b⁺F4/80⁺CD11c⁺) was significantly increased in HFD-fed *Gnpat*⁺/⁻ mice, and this effect was attenuated by AG treatment. The proportion of M2-like ATMs (CD11b⁺F4/80⁺CD301⁺) remained unchanged across all groups. Data are presented as mean ± SEM (n=5). ^**^P < 0.01, ^***^***P*** < 0.005; ^##^P < 0.01, ^###^P < 0.005; n.s., not significant.

**Supplementary Figure 9: AG suppresses LPS-induced M1 polarization without promoting M2 macrophage marker expression.**

(A) Relative mRNA expression of M1-associated markers in J774 macrophages treated with control (Con), LPS alone, or LPS with AG (LPS+AG). (B) Relative mRNA expression of M2-associated markers in the same treatment groups. Data are expressed as mean ± SEM (n=5). ^*^*P* < 0.05 and ^***^*P* < 0.005 versus the Con group; ^#^*P* < 0.05 and ^###^*P* < 0.005 versus the LPS group; n.s., not significant.

**Supplementary Figure 10: GNPAT overexpression modulates PE- and PC-plasmalogen abundance in LPS-stimulated macrophages.**

(A) Relative *Gnpat* mRNA expression in J774 macrophages transfected with a control vector (Vector) or a *Gnpat* overexpression vector (*Gnpat* OE). (B) Abundance of the indicated PE-plasmalogen molecular species in control, LPS-stimulated, and LPS-stimulated GNPAT-overexpressing J774 macrophages. (C) Abundance of the indicated PC-plasmalogen molecular species under the same experimental conditions. Plasmalogen abundance was normalized to protein content and expressed as pmol/ng protein. Data are presented as mean ± SEM (n = 3). ^**^*P* < 0.01 and ^***^*P* < 0.005 versus control; ^#^*P* < 0.05 and ^##^*P* < 0.01 versus LPS-stimulated cells; ns, not significant.

**Supplementary Figure 11: GNPAT overexpression does not affect JNK or NF-κB signaling pathways.**

Immunoblot analysis of phosphorylated IκBα and JNK in J774 macrophages overexpressing GNPAT (*Gnpat* OE) or transfected with a control vector (Vec) after LPS stimulation.

**Supplementary Figure 12: GNPAT overexpression suppresses LPS-induced *Ccl2* promoter activity through a STAT1-responsive regulatory element.**

*Ccl2* promoter-driven luciferase activity was measured in J774 macrophages using a mouse *Ccl2* promoter construct (−458 to +1 bp) containing a STAT1-binding regulatory element. Cells were co-transduced with *Ccl2* promoter lentivirus and GNPAT-overexpressing (GNPAT O/E) lentivirus at the indicated MOIs (5 and 25), followed by stimulation with 5 ng/mL LPS for 24 h. Data are presented as mean ± SEM (n = 3). ****P* < 0.005 versus the untreated Vector group; ^##^P < 0.01 and ^###^P < 0.005 versus the LPS-treated Vector group.

**Supplementary Figure 13: Modulation of macrophage inflammatory activation preserves insulin signaling in adipocytes through a paracrine mechanism.**

(Left) Representative immunoblot analysis of P-AKT and T-AKT in differentiated 3T3-L1 adipocytes exposed to macrophage-conditioned medium followed by insulin stimulation. (Right) Quantification of the P-AKT/T-AKT ratio. Data are presented as mean ± SEM (n = 3). ^***^*P* < 0.005 versus the control group (Vec + Ins + CM Con); ^#^*P* < 0.05 versus the LPS-treated macrophage-conditioned medium group with vector control (Vec + Ins + CM LPS).

**Supplementary Table 1. Primer list**

| **Gene** | **Forward sequence** | **Reverse sequence** |
| --- | --- | --- |
| CD11c | CTGGATAGCCTTTCTTCTGCTG | GCACACTGTGTCCGAACTCA |
| F4/80 | CTTTGGCTATGGGCTTCCAGTC | GCAAGGAGGACAGAGTTTATCGTG |
| *Il-6* | CCAGAGATACAAAGAAATGATGG | ACTCCAGAAGACCAGAGGAAAT |
| iNOS | TCATGACATCGACCAGAAGC | GGACATCAAAGGTCTCACAG |
| *Ccl2* | TTAAAAACCTGGATCGGAACCAA | GCATTAGCTTCAGATTTACGGGT |
| *Tnf* | GAGAAAGTCAACCTCCTCTCTG | GAAGACTCCTCCCAGGTATATG |
| *Arg1* | CTCCAAGCCAAAGTCCTTAGAG | AGGAGCTGTCATTAGGGACATC |
| CD163 | CCTTGGAAACAGAGACAGGC | TCCACACGTCCAGAACAGTC |
| CD206 | TGATTACGAGCAGTGGAAGC | GTTCACCGTAAGCCCAATTT |
| *Il-10* | TAGAGCTGCGGACTGCCTTCA | ATGCTCCTTGATTTCTGGGCCAT |
| *Gnpat* | TCATGCGACGCACTTTTGGA | TCCCTTCGAGGAAAAATTCAACA |
| *Fasn* | GGAGGTGGTGATAGCCGGTAT | TGGGTAATCCATAGAGCCCAG |
| *Acly* | CAGCCAAGGCAATTTCAGAGC | CTCGACGTTTGATTAACTGGTCT |
| *Srebp1c* | GATGTGCGAACTGGACACAG | CATAGGGGGCGTCAAACAG |
| *Cpt1a* | TGGCCGCATGTCAAGCCAGA | AGGAGAGCAGCACCTTCAGC |
| *Atgl* | CAACGCCACTCACATCTACGG | GGACACCTCAATAATGTTGGCAC |
| *Hsl* | AAGGACTTGAGCAACTCAGA | TTGACTATGGGTGACGTGTA |
| *Tbp* | CCTTCACCAATGACTCCTATGAC | CAAGTTTACAGCCAAGATTCAC |
